# Sex Differences in Social, Health, and Lifestyle Characteristics Associated With Binge‐Eating Behaviors: Results From a French National Random Population‐Based Study

**DOI:** 10.1002/eat.70113

**Published:** 2026-04-29

**Authors:** Junko Kose, Nathalie Godart, Anne Pastorello, Caroline Huas, Alexandra Rouquette, EpiCov Study Group, Josiane Warszawski, Nathalie Bajos, Guillaume Bagein, François Beck, Emilie Counil, Florence Jusot, Nathalie Lydié, Claude Martin, Laurence Meyer, Philippe Raynaud, Alexandra Rouquette, Ariane Pailhé, Delphine Rahib, Patrick Sillard, Rémy Slama, Alexis Spire

**Affiliations:** ^1^ Université Paris‐Saclay, Inserm Université Versailles Saint‐Quentin (UVSQ), Centre de recherche en Epidémiologie et de Santé Publique (CESP) Paris France; ^2^ Fondation de Santé des Etudiants de France (FSEF) Paris France; ^3^ Université Paris‐Cité, Inserm Centre of Research in Epidemiology and StatisticS, METHODS Team Paris France; ^4^ Department of Family Medicine, Faculty of Health Sciences Simone Veil Université Versailles‐Saint‐Quentin‐en‐Yvelines (UVSQ) Montigny le Bretonneux France; ^5^ Epidemiology and Public Health Department Assistance Publique‐Hôpitaux de Paris Université Paris‐Saclay Le Kremlin‐Bicêtre France

**Keywords:** binge eating, lifestyle, national random sample, public health, sex differences, social characteristics, somatic and mental conditions

## Abstract

**Objective:**

To explore sex differences in social, health and lifestyle characteristics associated with binge‐eating behaviors in a large population‐based study.

**Method:**

This study included 84,995 participants (women 52.1%) aged ≥ 15 years from the French national random population‐based EpiCov cohort. We assessed binge‐eating (BE) behaviors (No BE; BE without compensatory behavior [BE−]; BE with a compensatory behavior [BE+]) using the Patient Health Questionnaire Eating Disorder module in 2021. After testing interactions between exposures (self‐reported social, health and lifestyle characteristics) and sex for BE behaviors, weighted multinomial logistic regressions were performed to explore the associations between exposures and BE behaviors stratified by sex.

**Result:**

The prevalence of BE− and BE+ was higher among women (3.4 [3.2–3.7]% and 1.2 [1.1–1.3]%, respectively) than among men (2.1 [1.9–2.3]% and 0.7 [0.6–0.8]%, respectively). Sex modified the associations that were observed between BE− and poor perceived health status (adjusted odds ratio [95% CI]: men 2.43 [1.90–3.09], women 1.53 [1.29–1.80], interaction *p* = 0.008), and between BE+ and obesity (vs. normal weight; men 6.15 [3.92–9.64], women 2.15 [1.54–3.01], interaction *p* = 0.002). No other effect modification of sex was observed.

**Discussion:**

In this large study based on a national random sample from the French general population, the prevalence of binge eating was higher in women than in men. However, the associations between BE− and perceived health status, and between BE+ and obesity, were greater in men than women. The results highlight the need for targeted prevention strategies for BE accounting for sex differences.

## Summary

- The association between binge‐eating behaviors and poor health and that with obesity was stronger in men than in women in a random sample of the French population.
- The results highlight the need for targeted prevention strategies for binge eating accounting for sex differences.

**Abbreviations**
aOR: adjusted odds ratio
BE−: binge eating without compensatory behaviors
BE+: binge eating with a compensatory behavior
BED: binge eating disorder
BMI: body mass index
BN: bulimia nervosa
CI: confidence interval
DSM‐5: Diagnostic and Statistical Manual of Mental Disorders—5th edition
EDs: eating disorders
HRQOL: health‐related quality of life
PHQ‐ED: Patient Health Questionnaire—Eating Disorder

## 1 Introduction

Binge eating is characterized as recurrent consumption of unusually large amount of food in a discrete period of time (e.g., within 2‐h period), with a sense of lack of control over eating during the episode and feelings of guilt and distress linked to binge eating (American Psychiatric Association 2013). Although both binge‐eating disorder (BED) and bulimia nervosa (BN) exhibit regular binge‐eating episodes, compensatory behaviors to avoid weight gain following binge eating, such as self‐induced vomiting or excessive use of diuretic or laxatives, are less frequent in the case of BED than BN. Indeed, the Diagnostic and Statistical Manual of Mental Disorders—5th edition (DSM‐5) specifies that individuals with BN exhibit these compensatory behaviors at least once a week for 3 months, whereas BED is not associated with such regular behaviors. In consequence, BED is generally associated with obesity and extreme obesity, while people with BN often maintain their body weight at normal level (American Psychiatric Association 2013). According to the Global Burden of Disease study 2019, the prevalence of BED was higher than that of BN, with 223.4 for BED and 176.2 for BN and Anorexia Nervosa per 100 000 people (Santomauro et al. 2021). It is well studied that people suffering from BED frequently present various physical and mental conditions. Commonly co‐occurring conditions include obesity, hypertension, heart diseases, arthritis, elevated cholesterol and triglycerides, diabetes mellitus, gastrointestinal symptoms, respiratory problems, musculoskeletal problems, sleep problems, mood disorders, post‐traumatic stress disorder, anxiety disorders, and alcohol use disorders (Appolinario et al. 2022; Giel et al. 2022). As regards social characteristics, it has been reported that BED is prevalent in all socioeconomic groups and that those who have experienced poverty, violence, traumatic events, or food insecurity are at increased risk of BED (Giel et al. 2022). While investigating characteristics associated with binge‐eating behaviors will help to identify people at risk, most studies on eating disorders (EDs) have relied on clinical or convenience samples rather than representative ones (Giel et al. 2022; Guerdjikova et al. 2019). This may lead to underestimation of associated characteristics and limit the generalizability of the results to other populations, since individuals with binge‐eating behaviors who do not seek medical care may still be present in the community, and people with mental health issues are less likely to participate in epidemiological studies (Bu 2022). Studies on binge‐eating behaviors using epidemiological tool in general population will help to identify associated characteristics that inform primary prevention and intervention strategies.

While the prevalence of both BED and BN is higher in women than in men, the sex ratio of prevalence is more balanced for BED than for BN (6:4 and 9:1, respectively) (Guerdjikova et al. 2019). Studies suggest that sex disparities in EDs may partly reflect biological differences between sexes (e.g., correlation between estradiol and progesterone levels and disordered eating) and/or the influence of sex‐specific societal body ideals (e.g., thinness for women and muscularity for men) (Gorrell and Murray 2019; Guerdjikova et al. 2019). Among men, commitment to a diet characterized by targeted protein consumption and extreme dietary restriction and exercise may be motivated by the desire to achieve a body ideal in terms of muscularity, leading to muscularity‐oriented EDs (Gorrell and Murray 2019). By contrast, studies suggested that high‐caloric or high palatable foods (e.g., rich in sugars and fats) may be associated with greater binge eating frequency principally in women (Bjorlie et al. 2022; Guerdjikova et al. 2019). Additionally, an extensional study of the Global Burden of Diseases Study 2019 found differences in the age‐related pattern of EDs prevalence between sexes and across binge eating subtypes. Among women, BN was found to peak in the early to mid‐20s, whereas BED peaked in the late 20s to early 30s. In contrast, this age‐related variability was less pronounced in men, with both BN and BED showing peak prevalence in the late 20s to early 30s (Santomauro et al. 2021). Likewise, a nationally representative Norwegian longitudinal study (1992–2005) showed that binge eating in girls‐women decreased more significantly over time than in boys‐men, and that women are significantly more at risk of purging than boys‐men (Abebe et al. 2012). Moreover, some studies in small clinical settings (Guerdjikova et al. 2019) observed that binge‐eating behaviors in women were more likely to be associated with negative emotions such as anger, frustration, and anxiety than in men. Men with BED were more likely than women to have metabolic syndromes (high BMI and blood pressures) and to use medications for diabetes and dyslipidemia. No differences were observed in eating disturbance, interpersonal problems, or self‐esteem between sexes (Guerdjikova et al. 2019). Another study in a clinical sample with BED reported that women had significantly higher eating‐disorder psychopathology such as restraint, eating concern, shape concern, and weight concern than men, but no difference was observed in the frequency of binge‐eating episode between sexes (Lydecker and Grilo 2021). Despite the plausible hypotheses on the differences between the sexes, most studies on EDs were conducted in female and/or clinical samples, which lead to feature the profile of EDs from women implying that EDs manifest similarly in men and women (Gorrell and Murray 2019). An Australian population‐based study has however been conducted using 3034 representative male and female samples, revealing that association between objective binge eating and low mental health‐related quality of life (HRQOL) was stronger in men compared to women, and the association between overevaluation of body weight or shape and general and mental HRQOL was stronger in women than men (Mitchison et al. 2013). Yet, beyond these findings, evidence regarding sex differences across other core features of binge eating in the general representative population remains scarce. Research into sex‐differentiated binge‐eating behaviors in general population is therefore needed in order to provide new knowledge to this area. The aim of the present study was to explore whether characteristics associated with binge‐eating behaviors, including social, lifestyle, and health status, differed by sex in a random sample from French general population to emphasize the importance of raising awareness and implementing targeted hypothesis and future intervention research that could be tailored to sex.

## 2 Method

### 2.1 Study Population

The EpiCov (*Epidemiologie et conditions de vie sous le COVID‐19*) cohort was initiated by randomly selecting 371,000 participants aged ≥ 15 years in May 2020 and living in Metropolitan France or three overseas territories (Martinique, Guadeloupe, and Reunion) from the national tax database (*Fichier démographique sur les logements et les individus*—FIDELI database, coverage rate 96.4%). In order to ensure adequate statistical power to investigate social disparities, the sample frame was designed to overrepresent disadvantaged populations. Due to difficulties in reaching people living in medical nursing homes or prisons during the covid‐19 pandemic, they were not invited to participate in the study (Warszawski et al. 2022). Each participant was randomly assigned to either self‐computer‐assisted web interviews (CAWIs) or computer‐assisted telephone interviews (CATIs) to collect the data. The 134,391 participants at baseline were followed in Autumn 2020 (*N* = 107,759), in Summer 2021 (*N* = 85,074), and in Autumn 2022 (*N* = 65,423). The present cross‐sectional analysis is based on the 85,074 participants in the second follow‐up, which focused on mental health and took place between Jun 24 and August 6, 2021.

All participants provided informed consent to participate in the EpiCov study, which received approval from an ethics committee (*Comité de Protection des Personnes Sud Méditerranée* III 2020‐A01191‐38) and from France's National Data Protection Agency (*Commission Nationale Informatique et Libertés*, CNIL, MLD/MFI/AR205138).

### 2.2 Data Collection

#### 2.2.1 Outcomes: Binge Eating With and Without Compensatory Behaviors

Binge‐eating behaviors were assessed using the Patient Health Questionnaire—Eating Disorder (PHQ‐ED) (Striegel‐Moore et al. 2010). This questionnaire asks the respondent if he/she “often feels that he/she cannot control what he/she eats or how much he/she eats” and if: “he/she often eats, over a two‐hour period, a large amount of food that most people would consider abnormal.” If the person answered yes to either of these questions, further questions were asked about the frequency of binge eating and about any compensatory behaviors to avoid weight gain (vomiting; fasting for at least 24 h; exercising for more than an hour specifically to avoid gaining weight after eating too much; or taking more than twice the recommended dose of laxatives). If the respondent declares having such binge‐eating behaviors on average twice a week over the last 3 months, without and with compensatory mechanisms, he/she was categorized in “binge eating without compensatory behaviors: BE−” (proxy of BED) and “binge eating with a compensatory behavior: BE+” (proxy of BN) groups, respectively. Otherwise, the respondent was categorized in “no binge eating: No BE” group.

#### 2.2.2 Sex and Exposures

Data on sex (women vs. men) was provided by the FIDELI database, which corresponds to sex assigned at birth. On the basis of prior evidence, the following self‐reported social and lifestyle characteristics and health conditions well‐known to be associated with binge‐eating behaviors (Appolinario et al. 2022; Giel et al. 2022; Treasure et al. 2020; Udo and Grilo 2018) were selected as exposures: age category (17–25; 26–45; 46–64 vs. ≥ 65 years), educational attainment (> high school; = high school vs. < high school), employment status (paid or unpaid students; unemployed; other situation including retired, housewife or house husband, and others vs. employed), perceived financial difficulties (difficult, need to take loans vs. comfortable, decent, short), living alone (yes vs. no), immigration status (native or descendant from overseas France; second generation immigrant or above; first generation immigrant vs. metropolitan France), body mass index category (BMI, < 18.5; [25–30]; > 30 vs. [18.5–25]), perceived health status (very well to well vs. quite well to very poor), history of chronic somatic conditions (including respiratory diseases, hypertension, cardiovascular diseases, diabetes, digestive problems, gynecological problems, joint problems, rheumatism, cancer, HIV, immune problems, liver diseases, kidney diseases, physical handicap, and others; yes vs. no), history of diagnosed depressive disorders (yes vs. no), history of diagnosed anxiety disorders (yes vs. no), history of diagnosed other mental disorders (yes vs. no), suicidal ideation during last 12 months (yes vs. no), smoking status (former; current vs. never), alcohol use (occasional; often to daily vs. never), screen use > 6 h/d (yes vs. no), and physical activity at least 10 min/day, days/week (1–3 days; 4–7 days vs. 0 day). We included frequency of physical activity as an exposure because of plausible differences in compensatory behaviors between sexes (Gorrell and Murray 2019). The urban density of living area in 2018 (intermediate; high or Paris area vs. rural) was obtained from the FIDELI database.

#### 2.2.3 Covariates

We included the questionnaire type (CAWIs; CATIs) of the third wave to account for potential desirability bias due to the survey mode.

### 2.3 Statistical Analyses

**FIGURE 1:**
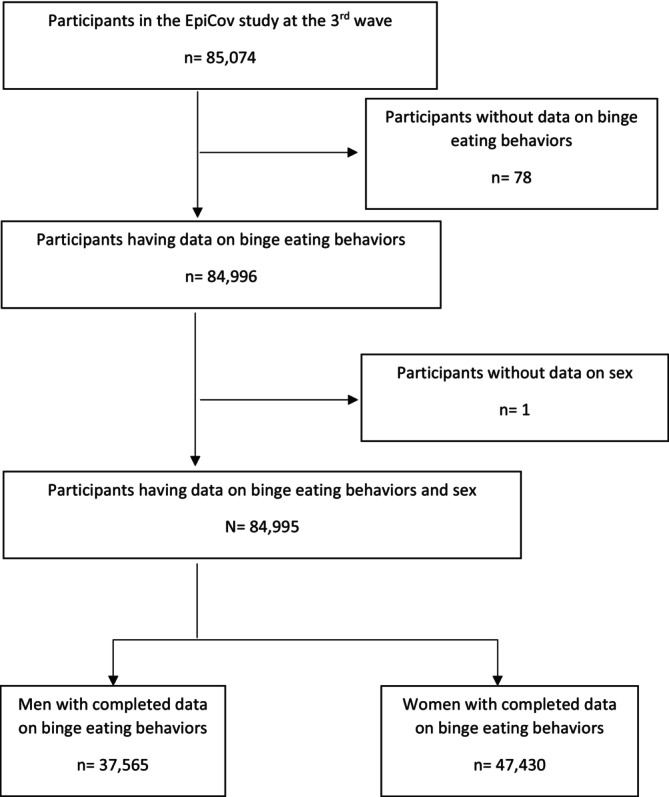
Flowchart of participants.

Participants with missing data on binge‐eating behaviors (*n* = 78) and sex (*n* = 1), which are the main variables of interest in the present study, were excluded from the analyses: a total of 84,995 participants was therefore included in this analysis (99.9% of respondents at the third wave) (Figure 1).

As preliminary analyses, multiplicative interactions between sex and each exposure on the association with binge‐eating behaviors were tested using multinomial logistic regression to identify interaction terms that would be included in the main model. Eighteen separate models were run, including all exposures, questionnaire type, and an interaction term between sex and each exposure (data not shown). These preliminary analyses were conducted to minimize multiplicity of tests in the main analyses by selecting interaction terms with a *p* value < 0.2 which allowed for a broader selection of potentially significant interactions to be retained in the subsequent adjusted model, while avoiding overly strict selection criteria.

For the main analyses, we first ran a single multinomial logistic regression model including all exposures, questionnaire type, and all interaction terms between sex and the exposures selected in the preliminary analyses, to identify significant interactions in the adjusted model (main model). Afterwards, we ran a multinomial logistic regression model including all exposures, thus mutually adjusted, and questionnaire type, stratified by sex to obtain adjusted odds ratios (aOR) according to sex.

In the sensitivity analysis, we repeated the same procedure by excluding exercises to prevent weight gain from compensatory behaviors to avoid conceptual overlap between exercises (outcome) and general physical activity frequency (exposure).

Data management was performed using SAS v9.4. EpiCov's survey design and non‐participation bias were taken into consideration by applying study weights (described elsewhere (Warszawski et al. 2021)) to descriptive, bivariate, and regression analyses. Briefly, survey weights were calculated using socioeconomic and demographic data from the FIDELI database and earlier EpiCov data collections relevant to predict non‐response. Questionnaire type (CAWI vs. CATI) was also included in the calculation of the study. Additionally, margins were adjusted to the general population using demographic projections and census data. Assuming that data were missing at random, multivariate imputation by chained equation (including all exposures, outcome, questionnaire type, and study weights; 10 imputations with 5 iterations) was used to handle missing data for exposures. Descriptive, bivariate (comparison of characteristics between sexes using Chi‐squared tests with Pearson's *X* ^2^ Rao and Scott adjustment), and regression analyses were performed using R 4.0. All tests were two‐sided and *p* < 0.05 was considered as evidence for statistical significance.

## 3 Results

### 3.1 Characteristics of the Participants

The characteristics of the included participants are presented according to sex (sociodemographic and economic status, questionnaire type: Table 1, health and lifestyle characteristics: Table 2). Among men, the prevalence of BE− and BE+ was 2.1 [1.9–2.3]% and 0.7 [0.6–0.8]%, respectively. Among women, the prevalence of BE− and BE+ was 3.4 [3.2–3.7]% and 1.2 [1.1–1.3]%, respectively. In bivariate analyses comparing the characteristics between sexes, men were more likely to be young, employed, report better perceived health, be overweight, smokers, and drinkers, perform physical activities, and less likely to live alone, to have financial difficulties, somatic conditions, and depressive and anxiety disorders, compared to women (Tables 1 and 2). Description of characteristics according to binge‐eating behaviors by sex is presented in the Tables S1 and S2.

**TABLE 1:** Sociodemographic characteristics of participants according to sex (*N* = 84,995; EpiCov study in 2021; weighted %; not imputed).

Characteristic	Overall^a^	Men^a^	Women^a^	*p* ^b^
	*N* = 89,995	*n* = 37,565	*n* = 47,430	
Age category				< 0.001
17–25 years	13.5% (9673)	14.5% (4138)	12.6% (5535)	
26–45 years	29.0% (23,423)	29.5% (9572)	28.5% (13,851)	
46–65 years	32.1% (33,350)	32.8% (14,876)	31.5% (18,474)	
More than 65 years	25.4% (18,549)	23.2% (8979)	27.4% (9570)	
Highest academic level^c^				0.103
< High school	46.7% (28,459)	47.0% (13,350)	46.5% (15,109)	
= High school	19.9% (17,372)	20.1% (7318)	19.7% (10,054)	
> High school	33.4% (39,005)	32.9% (16,817)	33.8% (22,188)	
Employment status^c^				< 0.001
Employed	46.4% (44,463)	49.8% (19,725)	43.3% (24,738)	
Paid or unpaid students	9.0% (7126)	9.3% (2996)	8.8% (4130)	
Unemployed	5.7% (3659)	6.2% (1598)	5.2% (2061)	
Other situation	38.9% (29,743)	34.8% (13,244)	42.6% (16,499)	
Perceived financial difficulties^c^				0.002
Hard or cannot make it without debt	11.1% (6702)	10.5% (2637)	11.6% (4065)	
Living alone^c^				< 0.001
Yes	21.8% (14,998)	20.4% (6130)	23.1% (8868)	
Urban density				0.270
Rural	22.4% (20,798)	22.7% (9287)	22.1% (11,511)	
Intermediate	61.6% (51,955)	61.6% (22,872)	61.7% (29,083)	
High—Paris area	15.9% (12,242)	15.7% (5406)	16.1% (6836)	
Immigration status^c^				0.111
Metropolitan France	76.6% (68,733)	76.5% (30,485)	76.7% (38,248)	
Native or descendant from France overseas	3.4% (2770)	3.3% (1140)	3.5% (1630)	
Second generation immigrant or above	9.6% (7142)	9.5% (3127)	9.7% (4015)	
First generation immigrant	10.5% (5194)	10.8% (2299)	10.1% (2895)	
Questionnaire type				0.018
Phone	22.1% (12,633)	22.7% (6005)	21.5% (6628)	
Web	77.9% (72,362)	77.3% (31,560)	78.5% (40,802)	

^a^ % (*n* (unweighted)).

^b^ Chi‐squared tests with Pearson's *X* ^2^ Rao and Scott adjustment for the comparison between sexes.

^c^ Number of missing values: Highest academic level 159, employment status 4, perceived financial difficulties 112, living alone 60, immigration status 1156.

**TABLE 2:** Health‐ and lifestyle‐related characteristics of participants according to sex (*N* = 84,995; EpiCov study in 2021; weighted %, not imputed).

Characteristic	Overall^a^	Men^a^	Women^a^	*p* ^b^
	*N* = 89,995	*n* = 37,565	*n* = 47,430	
Binge‐eating behaviors				< 0.001
Binge eating without compensatory behaviors	2.8% (2355)	2.1% (744)	3.4% (1611)	
Binge eating with compensatory behaviors	1.0% (792)	0.7% (271)	1.2% (521)	
Perceived health status^c^				< 0.001
Well to very well	74.3% (65,513)	77.4% (29,639)	71.5% (35,874)	
Quit well to very poor	25.7% (19,468)	22.6% (7921)	28.5% (11,547)	
Body Mass Index category^c^				< 0.001
< 18.5	3.6% (2959)	2.2% (650)	4.9% (2309)	
[18.5–25]	49.4% (43,271)	45.8% (16,963)	52.8% (26,308)	
[25–30]	30.9% (25,795)	36.7% (14,325)	25.6% (11,470)	
> 30	16.0% (12,329)	15.3% (5445)	16.7% (6884)	
History of chronic somatic conditions^c^				< 0.001
Yes	44.0% (36,690)	42.0% (16,240)	45.8% (20,452)	
History of diagnosed depressive disorders^c^				< 0.001
Yes	5.6% (5078)	4.4% (1616)	6.7% (3462)	
History of diagnosed anxiety disorders^c^				< 0.001
Yes	4.5% (3928)	3.7% (1312)	5.1% (2616)	
History of other diagnosed mental disorders^c^				0.588
Yes	4.1% (3280)	4.0% (1316)	4.1% (1965)	
Suicidal ideation during last 12 months^c^				0.060
Yes	2.8% (2314)	2.6% (932)	2.9% (1382)	
Smoking status^c^				< 0.001
Never smoker	47.9% (40,388)	40.4% (15,739)	54.9% (24,649)	
Former	29.6% (27,755)	34.5% (14,202)	25.0% (13,553)	
Smoker	22.5% (16,830)	25.1% (7611)	20.2% (9219)	
Alcohol use^c^				< 0.001
Never	26.5% (16,404)	19.8% (4982)	32.7% (11,422)	
Occasional	34.0% (30,361)	30.7% (11,516)	37.1% (18,845)	
Often to daily	39.5% (38,175)	49.5% (21,037)	30.3% (17,138)	
Screen use > 6 h/day^c^				0.068
Yes	16.7% (12,920)	17.1% (5781)	16.4% (7141)	
Physical activity > 10 min, days/week^c^				< 0.001
0 day	45.9% (34,380)	40.9% (13,617)	50.5% (20,763)	
1–3 days	29.2% (27,570)	30.4% (12,416)	28.1% (15,154)	
4–7 days	24.9% (22,415)	28.7% (11,297)	21.4% (11,118)	

^a^ % (*n* (unweighted)).

^b^ Chi‐squared tests with Pearson's *X* ^2^ Rao and Scott adjustment for the comparison between sexes.

^c^ Number of missing values: Perceived health status 14, body mass index category 641, history of chronic somatic conditions 43, history of diagnosed depressive disorders 494, history of diagnosed anxiety disorders 800, history of other mental disorders 3543, suicidal ideation 2357, smoking status 22, alcohol use 55, screen use 82, physical activity 630.

### 3.2 Sex Differences in Associated Characteristics With Binge‐Eating Behaviors

**FIGURE 2:**
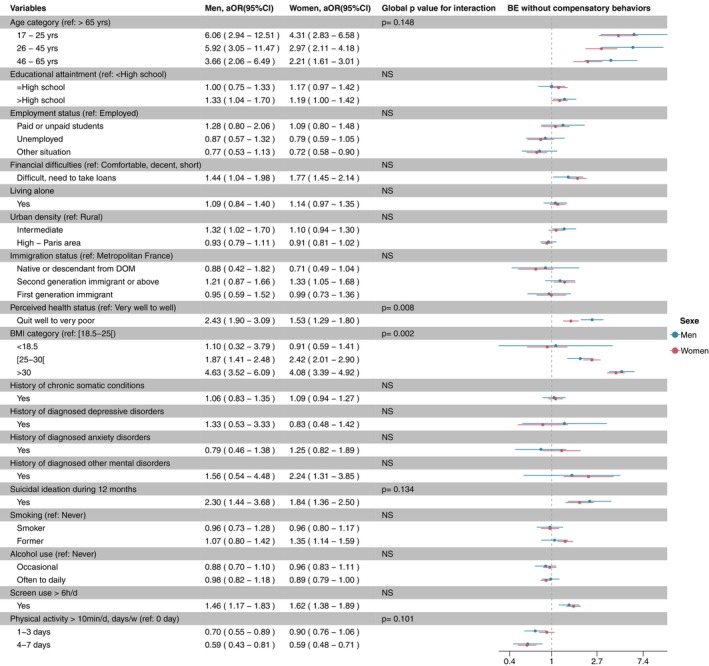
Sex differences in associations between binge eating without compensatory behaviors and social, lifestyle, and health factors (*N* = 84,995; EpiCov study in 2021; weighted and pooled after multivariate imputation by chained equation). *Note:* aOR: Adjusted odds ratio obtained from models stratified by sex, BE: Binge eating, CI: Confidence interval, NS: *p* ≥ 0.2 in preliminary analyses which were not included in the main model. The *p* values for interaction are identical in Figures 2 and 3, because they were derived from the same model (including all exposures, covariates and interaction terms with *p* < 0.2 in the preliminary analyses) assessing the association between each exposure and the outcome with three categories (No BE, BE−, BE+).

**FIGURE 3:**
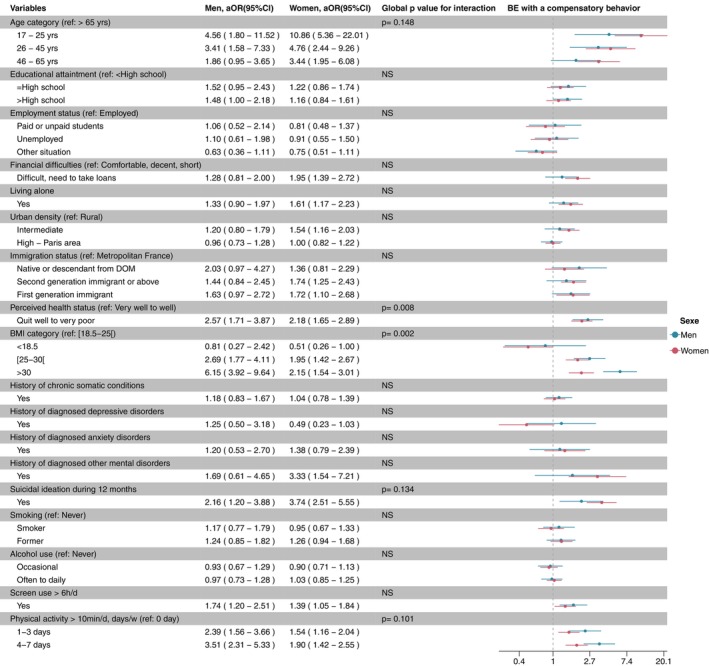
Sex differences in associations between binge eating with compensatory behaviors and social, lifestyle, and health factors (*N* = 84,995; EpiCov study in 2021; weighted and pooled after multivariate imputation by chained equation). *Note:* aOR: Adjusted odds ratio obtained from models stratified by sex, BE: Binge eating, CI: Confidence interval, NS: *p* ≥ 0.2 in preliminary analyses which were not included in the main model. The *p* values for interaction are identical in Figures 2 and 3, because they were derived from the same model (including all exposures, covariates and interaction terms with *p* < 0.2 in the preliminary analyses) assessing the association between each exposure and the outcome with three categories (No BE, BE−, BE+).

Adjusted associations between the studied characteristics and BE−, stratified by sex, are shown in Figure 2, and those for BE+ are shown in Figure 3. Modification effects of sex were observed for perceived health status (interaction *p* = 0.008) and BMI (interaction *p* = 0.002). As regards perceived health status, while participants with BE− were more likely to report poorer perceived health status than those with no BE in both sexes, the association was greater in men (aOR [95% confidence interval]: 2.43 [1.90–3.09]) than women (1.53 [1.29–1.80], Figure 2). Concerning BMI, while participants with BE+ were more likely to have a BMI > 30 than those with no BE in both sexes, the association was greater in men (6.15 [3.92–9.64]) than women (2.15 [1.54–3.01], Figure 3). No other significant modification effect of sex was observed in the main results.

For younger age, suicidal ideation, and screen use, the positive associations with both BE− and BE+ were similar in magnitude and direction for men and women, with overlapping confidence intervals. A similar pattern was observed for financial difficulties with BE−. Adjusted ORs [95% CI] varied from 1.74 [1.20–2.51] (screen use with BE+) to 6.06 [2.94–12.51] (age category 17–25 years with BE−) in men and from 1.39 [1.05–1.84] (screen use with BE+) to 10.86 [5.36–22.01] (age category 17–25 years with BE+) in women (interaction *p* values: age *p* = 0.148; suicidal ideation *p* = 0.134; financial difficulties and screen use *p* ≥ 0.2 in the preliminary analyses). Even though the modification effect of sex did not reach the significant level (interaction *p* = 0.101), the association between high frequency of physical activity (4–7 days/week) and BE+ seemed stronger in men (3.51 [2.31–5.33]) than in women (1.90 [1.42–2.55]) (Figure 3). In turn, high frequency of physical activity was inversely associated with BE− in a similar magnitude in both sexes (men 0.59 [0.43–0.81], women 0.59 [0.48–0.71], Figure 2). Other characteristics studied had no significant modification effect by sex and no significant association with binge‐eating behaviors in either or both sexes.

In the sensitivity analyses where exercises to avoid weight gain were excluded from compensatory behaviors, although similar results regarding sex differences were obtained across exposures, those for physical activity (exposure) were less pronounced in the sensitivity analyses compared to the main analyses (Figures S1 and S2).

## 4 Discussion

In this large study based on a national random sample from the French population in 2021, the prevalence of binge‐eating behaviors was higher in women than in men. Multinomial logistic regression analyses revealed that most factors studied were associated with binge‐eating behaviors in the same direction and magnitude between sexes. However, significant sex differences were observed: poor perceived health status was more strongly associated with BE− in men, while obesity showed a markedly stronger association with BE+ in men than in women.

In the present study, the prevalence of BE− and BE+ assessed using PHQ‐ED was 3.4% and 1.2% in women and 2.1% and 0.8% in men, respectively. A review study estimated that DSM‐5 based prevalence of BED was 1.5% in women and 0.3% in men worldwide (Keski‐Rahkonen 2021). Another review study reported that DSM‐5 based point prevalence of BN in both sexes varied from 0.1% to 0.8% across non‐clinical adult samples (Lindvall Dahlgren et al. 2017). These results align with the findings in a community‐based sample examining the operating characteristics, which reported higher prevalence assessed using PHQ‐ED than that estimated by clinical diagnosis (Striegel‐Moore et al. 2010). Indeed, studies have suggested that EDs are often under‐diagnosed due to factors such as stigma (Keski‐Rahkonen and Mustelin 2016). This highlights the importance of using epidemiological tools to study EDs in the general population, as they can help identify individuals exhibiting symptoms who may not seek professional consultation, thereby informing public health strategies and early intervention efforts.

Moderate positive association between poor perceived health status and BE− was observed in both sexes, and this association was more pronounced in men than in women. Because of the cross‐sectional study design, this result can be explained by sex differences in the perception of binge‐eating behaviors as a component of overall health status and/or variations in comorbid conditions associated with binge‐eating behaviors. While we identified no study on sex difference in perceived health status related to binge eating, an Australian population‐based study reported that objective binge eating had a stronger association with poor mental HRQOL in men compared to women (Mitchison et al. 2013). Another study in 562 men and women with BED reported that men exhibited elevated BMI values, increased systolic and diastolic blood pressure, and were prescribed a greater number of medications for managing diabetes and lipid disorders (Guerdjikova et al. 2014), which can be associated with poor perceived health status. However, we observed no difference between sexes for history of chronic somatic conditions in the present study. Further studies with other chronic diseases would elucidate the sex differences in this association. Another sex difference was observed for the association between obesity (BMI > 30) and BE+: strong positive associations between obesity and BE+ have been observed in both sexes, with substantially stronger association in men than in women, while no sex difference was observed for BE− and obesity in the present study. The results from a previous study in 9713 US college students were consistent with this findings: the odds of being overweight/obese compared to be normal weight seemed to be higher in men than women for the risk of ED (eating concern, shape concern, weight concern, and dietary restraint), but not for BED (Lipson and Sonneville 2017).

Younger age, suicidal ideation, and screen use each showed moderate to strong positive associations with both binge‐eating behaviors, without any significant modification effect by sex (i.e., no sex differences). In addition, both in men and women, financial difficulties and low frequency of physical activity were found to be moderately associated with BE− (positive association for financial difficulties and inverse association for frequency of physical activity), and high frequency of physical activity was found to be moderately and positively associated with BE+. Although we found no significant modification effect of sex on age, the association between younger age and BE− appeared stronger in men, whereas the association between young age and BE+ was more pronounced in women, according to point OR values. This result could mean that BE− and BE+ are more likely to occur in young age in men and women, respectively. Indeed, a study from the Global Burden of Disease Study 2019 reported that the prevalence of BN was higher in younger age compared to BED in women, while this pattern was not observed in men (Santomauro et al. 2021). As regards financial difficulties, a study in 158 female twins reported that binge eating may be heightened in those who faced financial difficulties even when accounting for potential genetic and environmental confounding factors (Mikhail et al. 2023). Another study suggested that food insecurity in early adolescence is associated with higher odds of developing future binge eating (Nagata et al. 2023), which may partially explain the association between financial difficulties and BE−. In our knowledge, the present study was the first to assess the association between screen use and binge eating in adult. Consistent with previous studies on the topic conducted in adolescents, we found that a high screen use was positively associated with both binge‐eating behaviors. This previous study in adolescents suggested that self‐esteem and depressive symptoms may play mediator role between this association (Al‐Shoaibi et al. 2024; Livet et al. 2024). Next, although high frequency of physical activity was significantly associated with BE+ in both sexes—given that exercise to avoid weight gain was among the compensatory behaviors assessed in the PHQ‐ED—no statistically significant sex difference was observed. However, the association appeared to be somewhat stronger in men. This may be due to the muscularity‐oriented nature of EDs in men, which could drive a greater frequency of physical activity as a compensatory behavior compared to women (Gorrell and Murray 2019). The results of sensitivity analyses support our hypothesis regarding the difference in compensatory behaviors between sexes. Finally, according to a review study, people suffering BN and BED had increased risk of suicide attempts than those without EDs (Attia and Walsh 2025), which aligns with the results in both sexes from our study.

No sex differences and non‐significant associations in either or both sexes were observed for educational attainment, employment status, living alone, immigration status, history of diagnosed depressive, anxiety, and mental disorders, smoking status, alcohol use for both BE behaviors, even though these factors were reported to be associated with binge‐eating behaviors (Attia and Walsh 2025; Giel et al. 2022; Guerdjikova et al. 2019; Keski‐Rahkonen 2021). This may due to the fact that previous studies were principally conducted in convenience study samples with fewer confounding factors considered in the associations, which prevent the generalizability of the study findings. Moreover, a review study concluded that the presentation of BED is largely comparable between males and females, with more similarities than differences (Guerdjikova et al. 2019), which were consistent with our results.

## 5 Limitations and Strengths

The limitations of the present study should be noted. First, the presence of symptoms defined by PHQ‐ED does not correspond to clinical definition of BN and BED, indicating the possibility of false positive detected by the PHQ‐ED. Indeed, the prevalence estimated by the PHQ‐ED is generally higher than that estimated by clinical diagnosis (Striegel‐Moore et al. 2010). Because of the self‐reported nature, potential misclassification between binge eating with and without compensatory behaviors is also plausible. In addition, PHQ‐ED based on the DSM‐IV criteria regarding the frequency of binge‐eating episode (at least twice a week), which lead to an underestimation of prevalence based on the DSM‐5 criteria (at least one a week). Further studies using DSM‐5 based clinical diagnosis as outcome could clarify the extent to which our findings generalize to clinically defined BN and BED. However, this 6‐item questionnaire presented high sensitivity (100%) and specificity (92%) in a community‐based sample (Striegel‐Moore et al. 2010) while minimizing burdens for participants. The use of an epidemiological tool in the general population allows to identify individuals exhibiting symptoms without professional consultation, which helps to inform primary prevention strategies of BN and BED. Second, we conducted a cross‐sectional study to identify associated characteristics with binge‐eating behaviors. This study design prevents any causal inference. Third, unmeasured confounders may exist, which could misestimate of the observed associations. In addition, selecting interaction terms based on preliminary analyses may introduce post‐selection inference bias, potentially leading to an overestimation of modification effects of sex in the main models. Our results need to be interpreted as hypothesis‐generating and to be confirmed in further studies. Fourth, we used data on sex from the FIDELI database, that is, sex assigned at birth, which does not allow us to study hypotheses related to gendered mechanisms other than strictly biological ones. Further studies including diverse gender identities would help provide new knowledge on this topic. Fifth, the exclusion of participants in nursing home and prison because of the difficulties in recruitment prevent to estimate the results in these populations. In addition, even though we applied weighting procedures to approximate estimates for the French general population, these remain estimations derived from our sample. We have no information on individuals who did not participate in the EpiCov study, which prevents us from assessing the accuracy of our estimates for non‐respondents. Finally, because of cultural and economic differences, our study would not be generalized beyond the French general population. Replication in other countries is needed to confirm these results.

Despite these limitations, this study has several important strengths. To our knowledge, this is the first large epidemiological study in the general population to explore sex differences in various characteristics associated with binge‐eating behaviors including various social, lifestyle, and health status. In addition, EpiCov participants were randomly selected from a national tax database covering more than 96% of the general population living in France. The strategy to overrepresent disadvantaged populations in recruitment along with study weights allowed us to address the fact that these populations are less likely to participate in epidemiological studies. Data were collected in 2021, representing the recent situation in France and weighted to account for non‐response, reducing potential selection bias.

## 6 Conclusion

This study in a large‐scale national random sample in France suggests that sex‐specific characteristics exist in binge‐eating behaviors. Although women had higher overall prevalence rates for both binge‐eating behaviors, the strength of associations between certain characteristics and binge‐eating behaviors was greater in men than in women. Specifically, poor perceived health status was more strongly associated with BE− in men, and obesity was more strongly associated with BE+ in men compared to women. Binge‐eating behaviors also had multifaceted characteristics regardless of sex, such as younger age, suicidal ideation, screen use, and physical activity frequency. This study indicated therefore the need for comprehensive public health strategies addressing BEDs in both men and women, moving beyond traditional female‐centric frameworks. Future research should further explore sex‐specific mechanisms and develop tailored interventions for BEDs across diverse populations.

## 7 Public Significance Statement

This study was the first to explore sex differences in social, health, and lifestyle characteristics associated with binge‐eating behaviors in a large, random population‐based sample. We found that the association between binge‐eating behaviors and poor health and that with obesity was stronger in men than in women. These findings emphasize the importance of raising awareness and implementing targeted hypotheses and future intervention research accounting for differences between sexes across the general population.

## Author Contributions

**Nathalie Godart:** conceptualization, methodology, supervision, writing – review and editing. **Alexandra Rouquette:** conceptualization, methodology, investigation, resources, supervision, project administration, writing – review and editing, funding acquisition. **Caroline Huas:** methodology, conceptualization, supervision, writing – review and editing. **Junko Kose:** conceptualization, methodology, formal analysis, visualization, project administration, writing – original draft. **Anne Pastorello:** methodology, writing – review and editing.

## Lived Experience Involvement Statement

No specific efforts were undertaken to involve persons with lived experience in the study design or execution, or in the preparation of this manuscript.

## Funding

This research was supported by the Institut National de la Santé et de la Recherche Médicale (INSERM), the French Ministry for Research, Direction de la Recherche, des Etudes, de l'Evaluation et des Statistiques (DREES), the French Ministry for Health, and the Région Ile de France. This project also received funding from the European Union's Horizon 2020 Research and Innovation Programme under grant agreement 101016167 ORCHESTRA (Connecting European Cohorts to Increase Common and Effective Response to SARS‐CoV‐2 Pandemic) (Pr Meyer), which contributed to the scientific development and allowed us to fund part of the data management.

## Ethics Statement

The studies involving human participants were reviewed and approved by Comité de Protection des Personnes Sud Méditerranée III 2020‐ A0119138 and France's National Data Protection Agency (Commission Nationale Informatique et Libertés, CNIL, MLD/MFI/AR205138). The patients/participants provided their written informed consent to participate in this study.

## Conflicts of Interest

The authors declare no conflicts of interest.

## Supporting information


**Figure S1:** Sex differences in associations between binge eating without compensatory behaviors and social, lifestyle, and health factors (*N* = 84,995; EpiCov study in 2021; weighted and pooled after multivariate imputation by chained equation); categorization of compensatory behaviors does not include exercises to avoid weight gain. *Note:* aOR: Adjusted odds ratio obtained from models stratified by sex, BE: Binge eating, CI: Confidence interval, NS: *p* ≥ 0.2 in preliminary analyses which were not included in the main model. The *p* values for interaction are identical in Figures 2 and 3, because they were derived from the same model (including all exposures, covariates and interaction terms with *p* < 0.2 in the preliminary analyses) assessing the association between each exposure and the outcome with three categories (No BE, BE−, BE+).

**Figure S2:** Sex differences in associations between binge eating with compensatory behaviors and social, lifestyle, and health factors (*N* = 84,995; EpiCov study in 2021; weighted and pooled after multivariate imputation by chained equation); categorization of compensatory behaviors does not include exercises to avoid weight gain. *Note:* aOR: Adjusted odds ratio obtained from models stratified by sex, BE: Binge eating, CI: Confidence interval, NS: *p* ≥ 0.2 in preliminary analyses which were not included in the main model. The *p* values for interaction are identical in Figures 2 and 3, because they were derived from the same model (including all exposures, covariates and interaction terms with *p* < 0.2 in the preliminary analyses) assessing the association between each exposure and the outcome with three categories (No BE, BE−, BE+).

**Table S1:** Characteristics of participants according to binge eating behaviors in men (*N* = 37,565; EpiCov study in 2021; weighted %; not imputed).
**Table S2:** Characteristics of participants according to binge eating behaviors in women (*N* = 47,430; EpiCov study in 2021; weighted %, not imputed).

## Data Availability

The non‐aggregated individual data cannot be shared publicly because of European Regulation 2016/679. Nonetheless, these data can be made available after submission to approval of French Ethics and Regulatory Committee procedure (Comité du Secret Statistique, CESREES and CNIL). The EpiCov dataset is available for research purposes on CASD (https://www.casd.eu/).
