# Supplementary material for: Sex Differences in Social, Health, and Lifestyle Characteristics Associated With Binge‐Eating Behaviors: Results From a French National Random Population‐Based Study
Source: Int J Eat Disord. 2026 Apr 29;59(8):1814–25. doi: 10.1002/eat.70113 (PMC13446237; doi:10.1002/eat.70113)

Variables	Men, aOR(95%CI)	Women, aOR(95%CI)	Global p value for interaction
Age category (ref: > 65 yrs)			p= 0.094
17 − 25 yrs	5.90 (3.14 − 11.08)	4.39 (2.94 − 6.55)	
26 − 45 yrs	5.32 (3.00 − 9.42)	3.04 (2.21 − 4.18)	
46 − 65 yrs	3.30 (2.02 − 5.40)	2.30 (1.72 − 3.07)	
Educational attaintment (ref: <High school)			NS
=High school	1.06 (0.81 − 1.38)	1.13 (0.94 − 1.36)	
>High school	1.36 (1.09 − 1.70)	1.14 (0.97 − 1.34)	
Employment status (ref: Employed)			NS
Paid or unpaid students	1.15 (0.75 − 1.75)	1.04 (0.78 − 1.40)	
Unemployed	0.95 (0.65 − 1.37)	0.82 (0.62 − 1.09)	
Other situation	0.72 (0.51 − 1.02)	0.73 (0.59 − 0.90)	
Financial difficulties (ref: Comfortable, decent, short)			NS
Difficult, need to take loans	1.36 (1.01 − 1.82)	1.79 (1.49 − 2.16)	
Living alone			NS
Yes	1.07 (0.84 − 1.35)	1.17 (0.99 − 1.37)	
Urban density (ref: Rural)			NS
Intermediate	1.24 (0.98 − 1.57)	1.20 (1.03 − 1.40)	
High − Paris area	1.31 (0.95 − 1.81)	1.19 (0.96 − 1.48)	
Immigration status (ref: Metropolitan France)			NS
Native or descendant from DOM	1.01 (0.55 − 1.87)	0.78 (0.56 − 1.10)	
Second generation immigrant or above	1.28 (0.96 − 1.72)	1.36 (1.10 − 1.70)	
First generation immigrant	1.00 (0.66 − 1.51)	1.05 (0.78 − 1.42)	
Perceived health status (ref: Very well to well)			p= 0.032
Quit well to very poor	2.42 (1.93 − 3.03)	1.55 (1.33 − 1.82)	
BMI category (ref: [18.5−25[)			p= 0.013
<18.5	1.05 (0.33 − 3.27)	0.85 (0.56 − 1.28)	
[25−30[	2.04 (1.59 − 2.63)	2.28 (1.92 − 2.71)	
>30	4.93 (3.82 − 6.34)	3.69 (3.09 − 4.40)	
History of chronic somatic conditions			p= 0.490
Yes	1.05 (0.84 − 1.31)	1.09 (0.94 − 1.26)	
History of diagnosed depressive disorders			NS
Yes	1.37 (0.61 − 3.08)	0.74 (0.44 − 1.26)	
History of diagnosed anxiety disorders			NS
Yes	0.89 (0.53 − 1.49)	1.27 (0.86 − 1.87)	
History of diagnosed other mental disorders			NS
Yes	1.52 (0.60 − 3.87)	2.46 (1.48 − 4.11)	
Suicidal ideation during 12 months			NS
Yes	2.05 (1.33 − 3.18)	1.87 (1.40 − 2.49)	
Smoking (ref: Never)			NS
Smoker	0.97 (0.75 − 1.25)	0.91 (0.76 − 1.10)	
Former	1.07 (0.84 − 1.38)	1.33 (1.14 − 1.56)	
Alcohol use (ref: Never)			NS
Occasional	0.97 (0.73 − 1.29)	1.08 (0.92 − 1.28)	
Often to daily	0.88 (0.66 − 1.17)	0.92 (0.76 − 1.11)	
Screen use > 6h/d			NS
Yes	1.52 (1.24 − 1.86)	1.56 (1.34 − 1.81)	
Physical activity > 10min/d, days/w (ref: 0 day)			NS
1−3 days	0.86 (0.69 − 1.06)	1.00 (0.86 − 1.17)	
4−7 days	0.90 (0.70 − 1.16)	0.80 (0.67 − 0.96)	

BE without compensatory behaviors

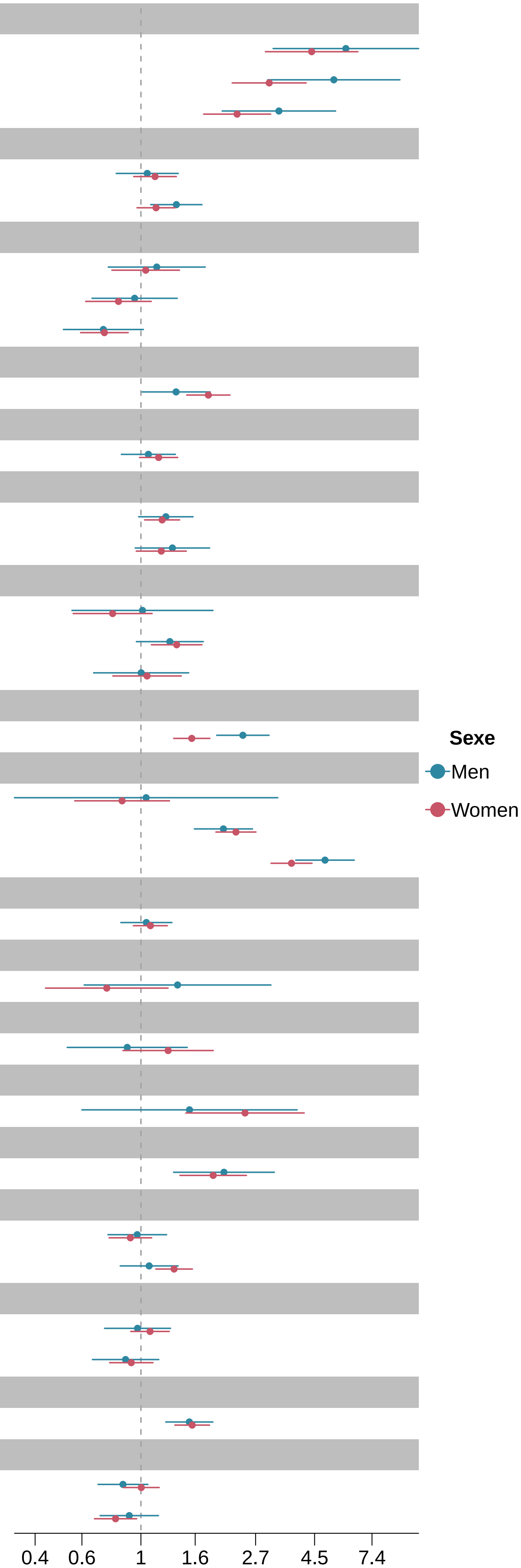

Supplement: Supplementary file 1 [file EAT-59-1814-s001.pdf]
