# Supplementary material for: Sex Differences in Social, Health, and Lifestyle Characteristics Associated With Binge‐Eating Behaviors: Results From a French National Random Population‐Based Study
Source: Int J Eat Disord. 2026 Apr 29;59(8):1814–25. doi: 10.1002/eat.70113 (PMC13446237; doi:10.1002/eat.70113)

Variables	Men, aOR(95%CI)	Women, aOR(95%CI)	Global p value for interaction
Age category (ref: > 65 yrs)			p= 0.094
17 − 25 yrs	4.36 (1.02 − 18.62)	19.18 (7.58 − 48.57)	
26 − 45 yrs	4.07 (1.28 − 12.95)	6.59 (2.57 − 16.89)	
46 − 65 yrs	1.67 (0.58 − 4.79)	3.98 (1.75 − 9.03)	
Educational attaintment (ref: <High school)			NS
=High school	1.80 (0.92 − 3.51)	1.50 (0.97 − 2.31)	
>High school	1.36 (0.73 − 2.55)	1.47 (0.95 − 2.27)	
Employment status (ref: Employed)			NS
Paid or unpaid students	1.80 (0.55 − 5.91)	0.86 (0.45 − 1.64)	
Unemployed	0.88 (0.36 − 2.17)	0.79 (0.46 − 1.35)	
Other situation	0.86 (0.39 − 1.93)	0.72 (0.42 − 1.24)	
Financial difficulties (ref: Comfortable, decent, short)			NS
Difficult, need to take loans	1.71 (0.93 − 3.14)	1.90 (1.28 − 2.84)	
Living alone			NS
Yes	1.99 (1.18 − 3.36)	1.84 (1.24 − 2.73)	
Urban density (ref: Rural)			NS
Intermediate	2.15 (0.99 − 4.67)	1.58 (1.06 − 2.34)	
High − Paris area	3.27 (1.37 − 7.82)	2.19 (1.31 − 3.67)	
Immigration status (ref: Metropolitan France)			NS
Native or descendant from DOM	2.53 (0.90 − 7.09)	1.34 (0.68 − 2.67)	
Second generation immigrant or above	1.07 (0.48 − 2.39)	1.82 (1.22 − 2.72)	
First generation immigrant	2.08 (1.02 − 4.25)	1.90 (1.11 − 3.26)	
Perceived health status (ref: Very well to well)			p= 0.032
Quit well to very poor	2.92 (1.71 − 5.00)	2.49 (1.74 − 3.57)	
BMI category (ref: [18.5−25[)			p= 0.013
<18.5	1.02 (0.22 − 4.78)	0.51 (0.22 − 1.19)	
[25−30[	2.26 (1.20 − 4.27)	2.24 (1.50 − 3.35)	
>30	5.73 (3.03 − 10.84)	2.57 (1.72 − 3.86)	
History of chronic somatic conditions			p= 0.490
Yes	1.53 (0.93 − 2.52)	1.07 (0.74 − 1.55)	
History of diagnosed depressive disorders			NS
Yes	0.87 (0.22 − 3.38)	0.57 (0.24 − 1.35)	
History of diagnosed anxiety disorders			NS
Yes	0.87 (0.31 − 2.46)	1.40 (0.72 − 2.72)	
History of diagnosed other mental disorders			NS
Yes	2.39 (0.61 − 9.44)	2.78 (1.08 − 7.17)	
Suicidal ideation during 12 months			NS
Yes	4.03 (1.95 − 8.32)	4.61 (2.87 − 7.41)	
Smoking (ref: Never)			NS
Smoker	1.61 (0.90 − 2.88)	1.24 (0.82 − 1.88)	
Former	1.56 (0.81 − 2.98)	1.29 (0.89 − 1.87)	
Alcohol use (ref: Never)			NS
Occasional	0.76 (0.39 − 1.47)	0.94 (0.65 − 1.36)	
Often to daily	0.64 (0.32 − 1.26)	0.91 (0.60 − 1.40)	
Screen use > 6h/d			NS
Yes	1.60 (0.89 − 2.86)	1.53 (1.09 − 2.15)	
Physical activity > 10min/d, days/w (ref: 0 day)			NS
1−3 days	1.70 (0.90 − 3.19)	1.13 (0.78 − 1.61)	
4−7 days	1.67 (0.84 − 3.30)	1.03 (0.71 − 1.49)	

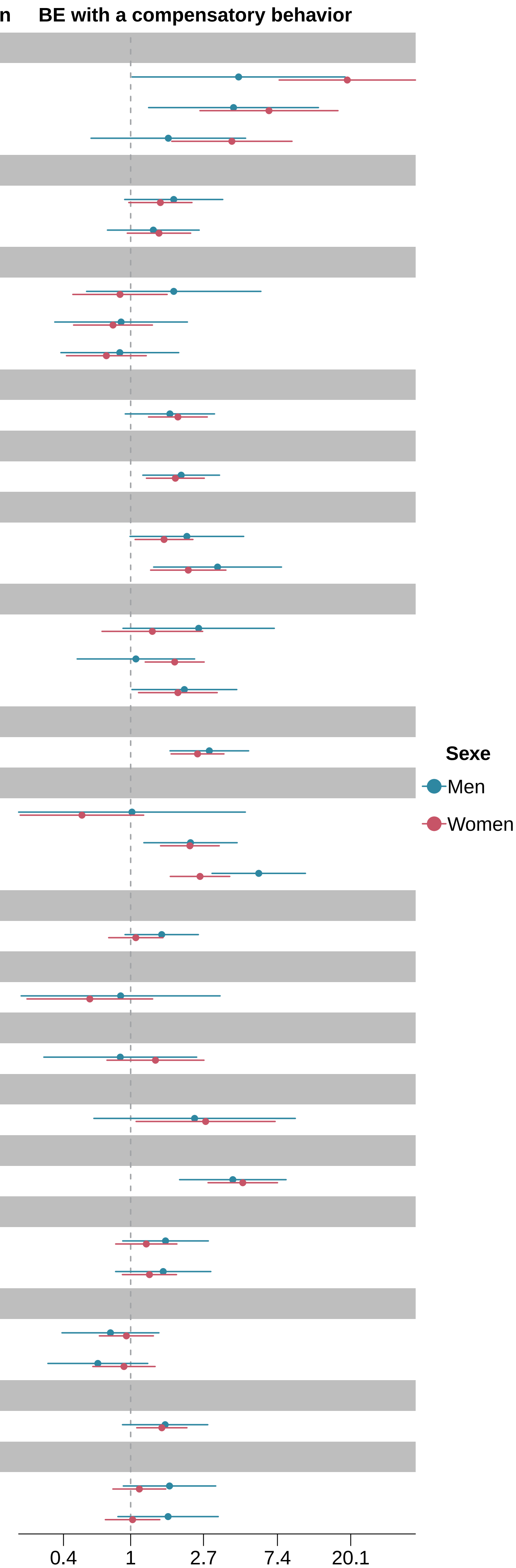

Supplement: Supplementary file 2 [file EAT-59-1814-s003.pdf]
