# Supplementary material for: Sex Differences in Social, Health, and Lifestyle Characteristics Associated With Binge‐Eating Behaviors: Results From a French National Random Population‐Based Study
Source: Int J Eat Disord. 2026 Apr 29;59(8):1814–25. doi: 10.1002/eat.70113 (PMC13446237; doi:10.1002/eat.70113)
Supplement: Supplementary file 3 [file EAT-59-1814-s002.docx]

**Supplementary Table S1**: Characteristics of participants according to binge eating behaviors in men (N= 37,565; EpiCov study in 2021; weighted %; not imputed)

| **Characteristic** | **No binge eating**^1^  97.2  [97.0-97.4] % | **Binge eating without compensatory behavior**^1^  2.1 [1.9-2.3] % | **Binge eating with compensatory behaivors**^1^  0.7 [0.6-0.8] % | **p-value**^2^ |
| --- | --- | --- | --- | --- |
| **Age category** |  |  |  | <0.001 |
| 15 - 25 yrs | 14.4% (3,995) | 16.9% (99) | 20.0% (44) |  |
| 26 - 45 yrs | 29.1% (9,171) | 42.9% (294) | 43.7% (107) |  |
| 46 - 65 yrs | 32.8% (14,478) | 34.4% (302) | 28.4% (96) |  |
| More than 65 yrs | 23.7% (8,906) | 5.8% (49) | 8.0% (24) |  |
| **Highest academic level^3^** |  |  |  | 0.001 |
| < High school | 47.2% (13,053) | 40.9% (221) | 34.7% (76) |  |
| = High school | 20.0% (7,107) | 20.1% (148) | 26.9% (63) |  |
| > High school | 32.7% (16,312) | 39.0% (373) | 38.3% (132) |  |
| **Employment status^3^** |  |  |  | <0.001 |
| Employed | 49.5% (19,075) | 58.9% (488) | 56.3% (162) |  |
| Paid or unpaid students | 9.2% (2,886) | 12.2% (77) | 13.4% (33) |  |
| Unemployed | 6.0% (1,522) | 9.4% (50) | 12.3% (26) |  |
| Other situation | 35.2% (13,065) | 19.5% (129) | 17.9% (50) |  |
| **Perceived financial difficulties^3^** |  |  |  | <0.001 |
| Difficult, need to take loans | 10.2% (2,478) | 21.7% (117) | 19.5% (42) |  |
| **Living alone^3^** |  |  |  | 0.005 |
| Yes | 20.2% (5,907) | 24.4% (159) | 28.9% (64) |  |
| **Urban density** |  |  |  | 0.008 |
| Rural | 22.9% (9,096) | 17.2% (145) | 16.7% (46) |  |
| Intermediate | 61.5% (22,222) | 63.0% (469) | 63.5% (181) |  |
| High - Paris area | 15.6% (5,232) | 19.8% (130) | 19.8% (44) |  |
| **Immigration status^3^** |  |  |  | 0.081 |
| Metropolitan France | 76.6% (29,704) | 74.2% (585) | 66.1% (196) |  |
| Native or descendant from France overseas | 3.3% (1,111) | 2.8% (17) | 6.3% (12) |  |
| Second generation immigrant or above | 9.4% (3,009) | 12.4% (87) | 13.0% (31) |  |
| First generation immigrant | 10.8% (2,233) | 10.6% (39) | 14.7% (27) |  |
| **Perceived health status^3^** |  |  |  | <0.001 |
| Well to very well | 77.9% (29,053) | 56.2% (413) | 59.1% (173) |  |
| Quit well to very poor | 22.1% (7,492) | 43.8% (331) | 40.9% (98) |  |
| **Body Mass Index catagory^3^** |  |  |  | <0.001 |
| <18.5 | 2.2% (641) | 2.2% (5) | 1.4% (4) |  |
| [18.5-25[ | 46.4% (16,730) | 26.0% (170) | 25.0% (63) |  |
| [25-30[ | 36.8% (13,943) | 33.0% (262) | 38.4% (120) |  |
| >30 | 14.7% (5,059) | 38.8% (303) | 35.2% (83) |  |
| **History of chronic somatic conditions^3^** | |  |  | 0.039 |
| Yes | 41.8% (15,737) | 47.5% (370) | 46.2% (133) |  |
| **History of diagnosed depressive disorders^3^** | |  |  | <0.001 |
| Yes | 4.2% (1,460) | 13.8% (114) | 14.7% (42) |  |
| **History of diagnosed anxiety disorders^3^** | |  |  | <0.001 |
| Yes | 3.5% (1,193) | 10.9% (85) | 13.5% (34) |  |
| **History of other diagnosed mental disorders^3^** | |  |  | <0.001 |
| Yes | 3.7% (1,188) | 12.6% (85) | 14.9% (43) |  |
| **Suicidal ideation during last 12 months^3^** | |  |  | <0.001 |
| Yes | 2.4% (835) | 10.4% (72) | 9.9% (25) |  |
| **Smoking status^3^** |  |  |  | 0.245 |
| Never smoker | 40.4% (15,334) | 39.6% (304) | 37.9% (101) |  |
| Smoker | 25.0% (7,362) | 29.1% (186) | 29.1% (63) |  |
| Former | 34.6% (13,842) | 31.3% (254) | 32.9% (106) |  |
| **Alcohol use^3^** |  |  |  | 0.005 |
| Never | 19.7% (4,800) | 24.9% (131) | 23.1% (51) |  |
| Occasional | 30.6% (11,163) | 34.0% (265) | 34.3% (88) |  |
| Often to daily | 49.7% (20,558) | 41.1% (347) | 42.6% (132) |  |
| **Screen use > 6h/d^3^** |  |  |  | <0.001 |
| Yes | 16.7% (5,505) | 31.3% (205) | 32.8% (71) |  |
| **Physical activity > 10 minutes, days/w^3^** | |  |  | <0.001 |
| 0 day | 40.6% (13,166) | 57.9% (390) | 25.5% (61) |  |
| 1-3 days | 30.5% (12,091) | 25.6% (234) | 35.4% (91) |  |
| 4-7 days | 28.9% (11,062) | 16.5% (117) | 39.1% (118) |  |
| **Questionnaire type** |  |  |  | 0.050 |
| Phone | 22.8% (5,874) | 17.1% (90) | 22.8% (41) |  |
| Web | 77.2% (30,676) | 82.9% (654) | 77.2% (230) |  |
| ^1^% (n (unweighted)) | | | | |
| ^2^Chi-squared tests with Pearson's X^2 Rao & Scott adjustment for the association between each factor and binge eating category (no BE, BE without compensatory behavior, and BE with compensatory behaviors) | | | | |
| ^3^ Number of missing values: Highest academic level 80, employment status 2, perceived financial difficulties 53, living alone 23, immigration status 514, perceived health status 5, body mass index category 182, history of chronic somatic conditions 15, history of diagnosed depressive disorders 232, history of diagnosed anxiety disorders 320, history of other mental disorders 1146, suicidal ideation 954, smoking status 13, alcohol use 30, screen use 32, physical activity 235. | | | | |

**Supplementary Table S2**: Characteristics of participants according to binge eating behaviors in women (N= 47,430; EpiCov study in 2021; weighted %, not imputed)

| **Characteristic** | **No binge eating**^1^  95.4  [95.1-95.6] % | **Binge eating without compensatory behavior**^1^  3.4 [3.2-3.7] % | **Binge eating with compensatory behaivors**^1^  1.2 [1.1-1.3] % | **p-value**^2^ |
| --- | --- | --- | --- | --- |
| **Age category** |  |  |  | <0.001 |
| 15 - 25 yrs | 12.2% (5,139) | 19.9% (277) | 27.5% (119) |  |
| 26 - 45 yrs | 28.1% (13,055) | 37.6% (611) | 36.0% (185) |  |
| 46 - 65 yrs | 31.5% (17,698) | 32.7% (594) | 30.4% (182) |  |
| More than 65 yrs | 28.3% (9,406) | 9.8% (129) | 6.2% (35) |  |
| **Highest academic level^3^** |  |  |  | <0.001 |
| < High school | 46.9% (14,534) | 38.9% (432) | 36.4% (143) |  |
| = High school | 19.4% (9,527) | 24.7% (402) | 26.1% (125) |  |
| > High school | 33.7% (21,159) | 36.4% (776) | 37.5% (253) |  |
| **Employment status^3^** |  |  |  | <0.001 |
| Employed | 43.0% (23,513) | 51.2% (941) | 49.3% (284) |  |
| Paid or unpaid students | 8.5% (3,851) | 13.6% (193) | 17.0% (86) |  |
| Unemployed | 5.1% (1,925) | 7.7% (100) | 9.8% (36) |  |
| Other situation | 43.4% (16,007) | 27.4% (377) | 23.9% (115) |  |
| **Perceived financial difficulties^3^** |  |  |  | <0.001 |
| Difficult, need to take loans | 10.9% (3,643) | 25.7% (302) | 29.1% (120) |  |
| **Living alone^3^** |  |  |  | 0.095 |
| Yes | 23.1% (8,402) | 22.5% (341) | 28.8% (125) |  |
| **Urban density** |  |  |  | <0.001 |
| Rural | 22.4% (11,053) | 18.7% (365) | 13.9% (93) |  |
| Intermediate | 61.6% (27,777) | 64.5% (982) | 60.5% (324) |  |
| High - Paris area | 16.0% (6,468) | 16.8% (264) | 25.6% (104) |  |
| **Immigration status^3^** |  |  |  | <0.001 |
| Metropolitan France | 77.0% (36,630) | 73.2% (1,261) | 61.8% (357) |  |
| Native or descendant from France overseas | 3.5% (1,550) | 3.0% (52) | 5.0% (28) |  |
| Second generation immigrant or above | 9.4% (3,753) | 14.2% (175) | 18.5% (87) |  |
| First generation immigrant | 10.1% (2,750) | 9.6% (102) | 14.7% (43) |  |
| **Perceived health status^3^** |  |  |  | <0.001 |
| Well to very well | 72.2% (34,622) | 58.6% (972) | 53.6% (280) |  |
| Quit well to very poor | 27.8% (10,667) | 41.4% (639) | 46.4% (241) |  |
| **Body Mass Index catagory^3^** |  |  |  | <0.001 |
| <18.5 | 5.0% (2,251) | 3.4% (44) | 2.8% (14) |  |
| [18.5-25[ | 53.8% (25,598) | 29.0% (471) | 39.7% (239) |  |
| [25-30[ | 25.4% (10,841) | 30.1% (488) | 32.2% (141) |  |
| >30 | 15.8% (6,165) | 37.4% (595) | 25.4% (124) |  |
| **History of chronic somatic conditions^3^** |  |  |  | 0.008 |
| Yes | 45.6% (19,379) | 50.8% (824) | 48.6% (249) |  |
| **History of diagnosed depressive disorders^3^** | |  |  | <0.001 |
| Yes | 6.1% (3,035) | 18.3% (297) | 20.9% (130) |  |
| **History of diagnosed anxiety disorders^3^** | |  |  | <0.001 |
| Yes | 4.6% (2,254) | 15.5% (243) | 19.1% (119) |  |
| **History of other diagnosed mental disorders^3^** | |  |  | <0.001 |
| Yes | 3.4% (1,589) | 17.2% (251) | 21.3% (125) |  |
| **Suicidal ideation during last 12 months^3^** | |  |  | <0.001 |
| Yes | 2.5% (1,161) | 9.6% (137) | 19.3% (84) |  |
| **Smoking status^3^** |  |  |  | <0.001 |
| Never smoker | 55.2% (23,652) | 46.6% (750) | 49.9% (247) |  |
| Smoker | 20.0% (8,763) | 24.0% (345) | 24.5% (111) |  |
| Former | 24.8% (12,874) | 29.4% (516) | 25.6% (163) |  |
| **Alcohol use^3^** |  |  |  | <0.001 |
| Never | 32.5% (10,832) | 34.3% (436) | 38.7% (154) |  |
| Occasional | 36.9% (17,906) | 40.9% (719) | 35.9% (220) |  |
| Often to daily | 30.5% (16,536) | 24.8% (455) | 25.4% (147) |  |
| **Screen use > 6h/d^3^** |  |  |  | <0.001 |
| Yes | 15.7% (6,543) | 31.9% (454) | 30.3% (144) |  |
| **Physical activity > 10 minutes, days/w^3^** |  |  |  | <0.001 |
| 0 day | 50.2% (19,653) | 61.6% (915) | 42.7% (195) |  |
| 1-3 days | 28.1% (14,523) | 27.2% (464) | 31.6% (167) |  |
| 4-7 days | 21.7% (10,745) | 11.3% (219) | 25.7% (154) |  |
| **Questionnaire type** |  |  |  | <0.001 |
| Phone | 21.9% (6,433) | 13.2% (152) | 14.2% (43) |  |
| Web | 78.1% (38,865) | 86.8% (1,459) | 85.8% (478) |  |
| ^1^% (n (unweighted)) | | | | |
| ^2^Chi-squared tests with Pearson's X^2 Rao & Scott adjustment for the association between each factor and binge eating behaviors (no BE, BE without compensatory behavior, and BE with compensatory behaviors) | | | | |
| ^3^Number of Missing values: Highest academic level 79, employment status 2, perceived financial difficulties 59, living alone 37, immigration status 642, perceived health status 9, body mass index category 459, history of chronic somatic conditions 28, history of diagnosed depressive disorders 262, history of diagnosed anxiety disorders 480, history of other mental disorders 2394, suicidal ideation 1403, smoking status 9, alcohol use 25, screen use 50, physical activity 395. | | | | |
